# The health behaviours of European study abroad students sampled from forty-two countries: Data from a three-wave longitudinal study

**DOI:** 10.1016/j.dib.2021.107285

**Published:** 2021-08-13

**Authors:** Giovanni Aresi, Simon C. Moore, Elena Marta

**Affiliations:** a Psychology Department, Università Cattolica del Sacro Cuore, Largo Gemelli 1, 20123 Milano, Italy; b CERISVICO Research Centre on Community Development and Organisational Quality of Life, via Trieste 17, 25121 Brescia, Italy; c Violence and Society Research Group, School of Dentistry, Cardiff University, Heath Park, Cardiff CF14 4XY, UK

**Keywords:** Study abroad, Credit mobility, Health behaviours, Social norms, Motivations to study abroad, Acculturation orientation, Psychological adjustment, Longitudinal design

## Abstract

Research on travelling populations indicates that geographic mobility is associated with changes in health behaviours. However, there is currently little longitudinal data recording study abroad students' health behaviours other than alcohol use [1], [2], and that includes a variety of risk and protective factors related to students' demographics and their experiences abroad.

The present dataset contains the original longitudinal data from a study of European study abroad students' and includes information on participants health-related behaviour: including physical exercise, diet, alcohol and drug use, and unprotected casual sex. Self-reported data were collected across three waves: on arrival in the host country, to assess pre-departure behaviour (T_1_), four months through the period abroad (T_2_), and four months after returning home (T_3_). Data on factors related to participants' demographics and their abroad experience, including motivations to study abroad, acculturation orientation and adjustment to the host environment, and perceptions regarding different referent peers’ drinking behaviour were also collected.

Data were collected in the 2015–2016 academic year. At T_1_ students in 200 cities from more than 40 European countries were approached by representatives from an international student association. Participants who completed at least two surveys were included (*N* = 908). The T_1_ survey was completed by 899 students (nine students provided an e-mail address but did not complete the survey at T_1_), 785 (86.5%) completed T_2_ survey, and 438 (48.2%) the T_3_ survey. The data article presents tables charting variables measured by survey wave and participants' socio-demographic and study abroad experience characteristics.

With an acceptable drop-out across the three waves, these data may be of interest to researchers who wish to understand factors related to changes in health behaviours in this population and develop targeted health promotion interventions. Other stakeholders such as policy makers, international offices, health professionals in counselling service, student associations may also use these data to develop communication campaigns and intervene with reference to relevant risk and protective factors.

## Specifications Table

**Table:** 

Subject	Social Sciences; Psychology; Public Health and Health Policy.
Specific subject area	Health behaviours.
Type of data	Tables, Excel and open document files.
How data were acquired	Data was gathered utilizing an online survey platform (qualtrics). See Data accessibility section to download the questionnaire.
Data format	Raw
Parameters for data collection	Respondents were eligible if they were participating in a study abroad programme, intended to stay abroad for a period of four months or more, and travelled from and to a European country.
Description of data collection	Data were collected in 2015–2016 academic year. We collected the data in cooperation with an international student organisation whose representatives approached study abroad students in 40 European countries. Participants completed the T_1_ online survey and provided their e-mail address. Participants were sent a link to the survey four months through the period abroad (T_2_) and four months after returning home (T_3_). Participants who participated in at least two waves were included (*N* = 908). Of these, the T1 survey was completed by 899 students (nine students provided an e-mail address but did not complete the survey at T_1_), 785 (86.5%) completed T_2_ survey, and 438 (48.2%) the T_3_ survey.
Data source location	Institution: Università Cattolica del Sacro Cuore City/Town/Region: Milan Country: Italy
Data accessibility	Repository name: The longitudinal health behaviours of European study abroad students sampled from forty-two countries and across three-waves. Data identification number: http://dx.doi.org/10.17632/585d2wdmtd.3 Direct URL to data: https://data.mendeley.com/datasets/585d2wdmtd/3
Related research article	Published articles: [3] G. Aresi, S. Alfieri, M. Lanz, E. Marta and S. Moore, 2018. Development and validation of a Multidimensional Motivations to Study Abroad Scale (MMSAS) among European Credit Mobility Students. International Journal of Intercultural Relations. 63: p. 128–134. 10.1016/j.ijintrel.2017.10.004. [4] G. Aresi, S.C. Moore, D.M. Berridge and E. Marta, 2019. A Longitudinal Study of European Students' Alcohol Use and Related Behaviors as They Travel Abroad to Study. Substance Use and Misuse. 54(7): p. 1167–1177. 10.1080/10,826,084.2019.1567787. The present dataset was co-submitted with: [5] G. Aresi, A. Sorgente, S.C. Moore and E. Marta, 2021. Analysing change among study abroad students. A Novel Application of the Person-Centred Approach to alcohol use patterns. International Journal of Intercultural Relations, 82, 220–231. 10.1016/j.ijintrel.2021.04.006

## Value of the Data

• Study abroad students are a growing population, making investigation on their health important from a public health perspective. This is the first study in this area tracking students as they move across Europe;
• The data will be useful for scientists interested in exploring the mechanisms underlying changes in a range of health behaviours (alcohol and drug use, unprotected casual sex, physical exercise, and diet) among students as they travel abroad and return to their home countries;
• The data can be analysed to feed into targeted health promotion intervention and policy development by researchers, policy makers, international offices, health professionals in counselling service, and student associations operating in host and home institutions;
• Longitudinal data concerning study abroad students' health behaviors other than alcohol use, that also combines a variety of risk and protective factors, are scarce. This allows for new insights in a field which is evolving, and where researchers and policy makers alike are looking for new insights;
• This dataset should inspire new substantive pieces of longitudinal research to analyse common patterns among health behaviours, as well as the effect of each risk/protective factor and in combination on behavioural patterns. Further analyses can be done considering the clustered nature of the data (i.e., home and host country), or to further refine measurement instruments and conduct country-specific analyses.

## 1 Data Description

The present dataset contains longitudinal raw data on European study abroad students' health behaviours and a number of risk/protective factors related to participants' demographics and their abroad experience. Data were collected as part of Lifestyle in Mobility, a collaborative research project among the Università Cattolica del Sacro Cuore (Italy), Cardiff University (UK) and the international student association AEGEE-Europe. The project aimed at investigating patterns of health-related behaviours among European study abroad students.

Data were collected three times: on arrival in the host country, though assessing pre-departure behaviour (T_1_), four months through the period abroad (T_2_), and four months after returning home (T_3_). Data are provided as a SPSS and Excel file with rows representing cases and columns indicating variables. Codebooks provided (see Data accessibility section) identify variables by position in the dataset, variable names, variable labels, value labels, and missing values and describe the number of valid case observations. Table 1 charts measured variables survey wave. Participants' socio-demographic and study abroad experience characteristics are displayed in Table 2.

**Table 1:** Demographic and study abroad experience variables, and longitudinal indicators of health behaviours measured by survey wave.

	T_1_	T_2_	T_3_
Gender	*		
Age	*		
Type of living area	*		
Living accommodation	*	*	
Socio-economic status	*		
Discipline of study	*		
Years in education	*		
Mother language(s)	*		
Education mother and father	*		
Home country	*		
Past experience abroad	*		
Study abroad programme	*		
Programme length	*		
Programme was obligatory	*		
Host country	*		
Host country living cost		*	
Hours of study		*	
Physical exercise	*	*	*
Fruit consumption	*	*	*
Vegetables consumption	*	*	*
Sweets consumption	*	*	*
Soft drinks consumption	*	*	*
Last year alcohol use	*		
Last month alcohol use	*	*	*
Alcohol use by day of the week (Monday through Sunday)	*	*	*
Alcohol use by beverage (beer, wine, liqueur, spirits, mixed drinks)	*	*	*
Binge drinking episodes	*	*	*
Drunkenness episodes	*	*	*
Alcohol negative consequences	*	*	*
Last month cannabis use	*	*	*
Last month other illicit drugs use	*	*	*
Last month unprotected casual sex	*	*	*
Motivations to study abroad	*		
Acculturation orientation		*	
Sociocultural adaptation		*	
Psychological adaptation		*	
Alcohol use descriptive norms (compatriot)	*		
Alcohol use injunctive norms (compatriot)	*		
Alcohol use descriptive norms (international)	*	*	
Alcohol use injunctive norms (international)	*	*	
Alcohol use descriptive norms (host country peer)		*	
Alcohol use injunctive norms (host country peer)		*

**Table 2:** Participants' socio-demographic and study abroad experience characteristics (number and%, unless otherwise specified) (*N* = 908).

Variable	N (%)
Gender (female) (Missing data: *N* = 2)	659 (72.6)
Mean age (*SD*) (No missing data)	22.2 (2.28)
Type of living area (No missing data)	
Urban	606 (66.7)
Suburban	208 (22.9)
Rural	94 (10.4)
Living accommodation in home country (Missing data: *N* = 1)	
Apartment with international/study abroad students only	54 (5.9)
Apartment with at least one local person	247 (27.2)
University residence hall/dormitory	158 (17.4)
Host family	6 (0.7)
Family of origin / relatives	384 (42.3)
Other	58 (6.4)
Socio-economic status (Missing data: *N* = 49)	
Very much better off	11 (1.2)
Much better off	70 (7.7)
Better off	239 (26.3)
About the same	418 (46.0)
Less well off	98 (10.8)
Much less well off	17 (1.9)
Very much less well off	6 (0.7)
Discipline of study (Missing data: *N* = 48)	
Humanities (Language studies)	102 (11.2)
Humanities non-language studies (e.g., History, Literature, Arts)	60 (6.6)
Social Sciences (e.g., Anthropology, Economics, Political science)	209 (23.0)
Natural Sciences (e.g., Biology, Chemistry)	101 (11.1)
Formal Sciences (e.g., Mathematics, Computer sciences)	34 (3.7)
Professions (e.g., Architecture and design, Business)	304 (33.5)
Other	50 (5.5)
Mean number of years in education (No missing data)	3.1 (1.371)
Mother language(s) (seven most prevalent) (Missing data: *N* = 1)	
German	138 (15.2)
Spanish	119 (13.1)
Italian	88 (9.7)
French	87 (9.6)
English	72 (7.9)
Dutch	60 (6.6)
Lithuanian	38 (4.2)
Education mother (Missing data: *N* = 50)	
Bachelor's degree	239 (26.3)
Master's degree or higher	221 (24.3)
High school or equivalent	185 (20.4)
Vocational/technical school	141 (15.5)
Elementary or middle school or lower	72 (7.9)
Education father (Missing data: *N* = 51)	
Bachelor's degree	251 (27.6)
Master's degree or higher	189 (20.8)
High school or equivalent	182 (20.0)
Vocational/technical school	171 (18.8)
Elementary or middle school or lower	64 (7.0)
Home country (seven most prevalent) (No missing data)	
Spain	128 (14.1)
Germany	90 (9.9)
Italy	89 (9.8)
France	61 (6.7)
United Kingdom	58 (6.4)
Netherlands	44 (4.8)
Lithuania	40 (4.4)
Past experience abroad (yes) (No missing data)	322 (35.5)
Study abroad programme (No missing data)	
Study abroad semester/year programme	855 (94.2)
Training or internship/language training programme	53 (5.8)
Programme length (No missing data)	
Between three months and six months	613 (67.5)
Between six months and one year	280 (30.8)
More than a year	15 (1.7)
Study abroad programme obligatory (yes)	140 (15.4)
Host country (seven most prevalent) (No missing data)	
Spain	116 (12.8)
Italy	83 (9.1)
United Kingdom	79 (8.7)
France	72 (7.9)
Germany	68 (7.5)
Belgium	50 (5.5)
Netherlands	48 (5.3)
Living accommodation in host country (Missing data: *N* = 133)	
University residence hall/dormitory	295 (32.5)
Apartment with international/study abroad students only	235 (25.9)
Apartment with at least one local person	186 (20.5)
Host family	13 (1.4)
Family of origin / relatives	7 (0.8)
Other	39 (4.3)

## 2 Experimental Design, Materials and Methods

### 2.1 Questionnaire development

A systematic literature review [2] guided the identification of relevant risk/protective factors. Factors related to participants' demographics (e.g., age), and constructs related to their motivations to study abroad and their abroad experience (e.g., acculturation orientation), as well as normative perceptions regarding peers’ drinking behaviour were included in the survey. To measure constructs, we used the validated version of each measure in English, when available. In light of methodological and psychometric limitations in the existing measures of motivations to study abroad prior departure, the Multidimensional Motivations to Study Abroad Scale (MMSAS) was developed and validated as part of the study [3]. All measures included in the survey were first developed in English and translated (and back translated for accuracy) by native speakers into Dutch, French, German, Italian, and Spanish. To ensure translated versions retained the original meaning, any incongruence between the original and each back translated English version was resolved through discussion.

### 2.2 Measures

*Demographics and study abroad information.* At T_1_, participants provided their gender, age, type of living area, living accommodation in the home country, mother language(s), socio-economic status, current area of study, number of years in education, parents' level of education, country of origin and destination, whether they had any significant previous experience in a foreign country, and information on the study abroad programme they were participating in, including the amount of time they planned to spend abroad, and whether it was an obligatory part of study curriculum. At T_2_, students were asked about characteristics of their living accommodation, perceptions of their host country's living costs compared to their home country, the number of hours a week they usually spend studying or doing assignments (not including attending classes).

*Physical exercise and food habits*. To assess the weekly frequency of intense physical exercise and food intake of fruit, vegetables, sweets and soft drinks containing sugar, measures drawn from the Health Behaviours in School Children survey were used (authorisation of use obtained on 15 of April 2015). Participants were asked how many hours a week they usually exercised so much that you get out of breath or sweat (1 = none to 7 = About 7 h or more), and the frequency they ate or drank each food or drink type on a Likert scale (1 = never to 7 = Every day more than once).

*Alcohol consumption*. Respondents were asked whether they consumed any alcohol during the previous 12 months and 30 days. In the case of affirmative answers, they were asked to indicate the number of drinks consumed per occasion and which day(s) of a typical week of a given 30-day period they drank any alcohol. A validated drinking-day beverage-specific quantity measure [6] along with a standard drink definition were used for all measures and included validated images of alcoholic beverages (containing 10 g of alcohol) [7]. The past 30-day frequency of heavy episodic drinking (HED) (i.e., consuming at least four/five standard drinks for women and men in one drinking session), and the frequency of drunkenness episodes (defined as staggering when walking, not being able to speak properly, vomiting or an inability to recall events during the drinking session were measured at each survey.

*Alcohol negative consequences*. The 24 yes/no Brief Young Adult Alcohol Consequences Questionnaire (BYAACQ) [8] was used to assess the number of negative consequences participants experienced over a 30-day period.

*Drug use and risky sexual behaviour*. Respondents were asked whether they used cannabis, any other psychoactive drugs (e.g., cocaine), and had unprotected casual sex during the given 30-day period. In the case of an affirmative answer, they were asked about the frequency they engaged in each behaviour on a Likert scale (1 = never to 7 = 40 times or more).

*Motivations to study abroad*. The 27-item Multidimensional Motivations to Study Abroad Scale (MMSAS) was used to assess the importance of nine different motivations to study abroad prior departure [3]. Respondents were prompted as follows: “Think about the reasons why you want to study abroad. How important is each one of the following motivations to you?”. Participants responded to a scale from one (not important at all) to five (very important). In the validation study [3], the MMSAS nine-factor solution was confirmed in all languages (English for both native and non-native English speakers, French, German, Italian, and Spanish) except Dutch. Configural and metric invariance, but not scalar invariance was demonstrated across the languages. The analyses also provided preliminary evidence of criterion-validity. Cronbach's alpha (α) values of sub-scales are as follows: Personal growth = 0.795, Academic = 0.810, Others’ expectations = 0.747, Foreign language = 0.775, Cross-cultural = 0.859, Get away = 0.859, Career = 0.857, Independency = 0.805, and Leisure = 0.801.

*Acculturation orientation and adaptation to the host environment*. Three scales developed by Demes and Geeraert [9] were used to measure students' perceptions of acculturation orientation toward the home and host culture (Brief Acculturation Orientation Scale, BAOS), sociocultural (Brief Sociocultural Adaptation Scale, BSAS), and psychological adaptation (Brief Psychological Adaptation Scale, BPAS) to the host country. In the validation study [9], these scales demonstrated construct validity, structural unidimensional validity, and good internal reliability for all languages used in this study except Dutch. The BAOS is an eight-item bi-dimensional scales that measures acculturation orientation toward the home country and the host country independently. Participants were prompted as follows: “Think about being in the host country. How much do you agree with the following sentences? When in the host country, it is important for me to…”, then asked to rate their agreement with four statements such as “It is important for me to have [home country] friends”. These four items were presented twice, once for the home country and again for the hosting country. Participants responded to a scale from one (strongly disagree) to seven (strongly agree). Cronbach's alpha (α) is equal to 0.792 and 0.771 for the home and host country BAOS, respectively. The 12-item BSAS was used to measure students’ psychological well-being as it relates to their adaptation to the host country (α = 0.851). Respondents were prompted as follows: “Think about living in [host country]. In the last two weeks, how often have you felt…” to items such as “out of place, like you don't fit into the [host country] culture”. Participants responded to a scale from one (never) to seven (always). The eight-item BPAS was used to measure students’ psychological well-being as it relates to their adaptation to the host country (α = 0.842). Respondents were prompted as follows: “Think about living in [host country]. In the last two weeks, how often have you felt…” to items such as “out of place, like you don't fit into the [host country] culture”. Participants responded to a scale from one (never) to seven (always). All items, except item 1 and 8, are reversed.

*Descriptive and injunctive drinking norms*. Perceptions regarding peers’ drinking behaviour were measured using different reference groups. At pre-departure (T_1_), respondents were asked to think of a typical fellow compatriot peer and a typical study abroad student studying in the same host country that they were travelling to. During the study abroad experience (T_2_), they were asked to think of a typical study abroad student studying in the same host country and a typical host country peer. For descriptive norms, participants were asked to estimate each referent's monthly HED frequency on a seven-point Likert scale (1 “never” to 7 “40 times or more”). For injunctive norms, students were asked to rate the perceived acceptability of three behaviours taken from the House Acceptability Questionnaire [10,11]. The behaviours were “becoming intoxicated at a party,” “missing a class because you are intoxicated or hangover,” and “becoming intoxicated on a weeknight”. Participants responded to a scale that ranged from one (not acceptable) to seven (very acceptable). Cronbach's alpha (α) values are as follows: T_1_ typical fellow compatriot peer = 0.823, T_1_ typical study abroad student studying in the same host country = 0.858, T_2_ typical study abroad student studying in the same host country = 0.866, and T_2_ typical host country peer = 0.806.

### 2.3 Data collection

The data presented in this article were collected in the 2015–2016 academic year. At T_1_ approximately 1,800 students (across both cohorts) in 200 cities from more than 40 European countries were approached by representatives from an international student association. Two cohorts were recruited, the first starting at the beginning of the first semester (September 2015) and the second at the beginning of the second semester (February 2016). Respondents were eligible if they were participating in a study abroad programme, intended to stay abroad for a period of four months or more, and travelled from and to a European country. Only those who were contacted within the first two weeks after arrival were asked to participate in the study. Participants completed the T_1_ online survey and provided their e-mail address. Participants were sent a link to the survey four months through the period abroad (T_2_) and four months after returning home (T_3_). Participants who completed at least two surveys were included (*N* = 908) and were offered entry into a lottery for flight vouchers as an incentive. The T1 survey was completed by 899 students (nine students provided an e-mail address but did not complete the survey at T_1_), 785 (86.5%) completed T_2_ survey, and 438 (48.2%) the T_3_ survey.

## Ethics Statement

Ethical approval was obtained from the Human Research Ethics Committee at the Universita’ Cattolica del Sacro Cuore (#16–15) for all aspects of the current research. Research was conducted in accordance with the Declaration of Helsinki and informed consent was obtained from all participants.

## CRediT Author Statement

**Giovanni Aresi:** Conceptualization, Methodology, Data curation, Writing – original draft; **Simon C. Moore:** Writing – original draft, Methodology Supervision, Writing – reviewing & editing. **Elena Marta:** Writing – original draft, Methodology Supervision.

## Declaration of Competing Interest

The authors declare that they have no known competing financial interests or personal relationships which have or could be perceived to have influenced the work reported in this article.

## References

[1] Aresi G., Moore S., Marta E., Cairns D. (2021). Youth mobility, mental health and risky behaviours. The Palgrave Handbook of Youth Mobility and Educational Migration.

[2] Aresi G., Moore S., Marta E. (2016). Drinking, drug use and related consequences among university students completing study abroad experiences: a systematic review. Subst. Use Misuse.

[3] Aresi G., Alfieri S., Lanz M., Marta E., Moore S. (2018). Development and validation of a Multidimensional Motivations to Study Abroad Scale (MMSAS) among European credit mobility students. Int. J. Intercult. Relat..

[4] Aresi G., Moore S.C., Berridge D.M., Marta E. (2019). A longitudinal study of European Students' alcohol use and related behaviors as they travel abroad to study. Subst. Use Misuse.

[5] Aresi G., Sorgente A., Moore S.C., Marta E. (2021). Analysing change among study abroad students. A novel application of the person-centred approach to alcohol use patterns. Int. J. Intercult. Relat..

[6] Bloomfield K., Hope A., Kraus L. (2013). Alcohol survey measures for Europe: a literature review. Drugs Educ. Prev. Policy.

[7] Kuntsche E., Labhart F. (2012). Investigating the drinking patterns of young people over the course of the evening at weekends. Drug Alcohol Depend..

[8] Kahler C.W., Hustad J., Barnett N.P., Strong D.R., Borsari B. (2008). Validation of the 30-day version of the brief young adult alcohol consequences questionnaire for use in longitudinal studies. J. Stud. Alcohol Drugs.

[9] Demes K.A., Geeraert N. (2014). Measures matter: scales for adaptation, cultural distance, and acculturation orientation revisited. J. Cross Cult. Psychol..

[10] Larimer M.E., Irvine D.L., Kilmer J.R., Marlatt G.A. (1997). College drinking and the Greek system: examining the role of perceived norms for high–risk behavior. J. Coll. Stud. Dev..

[11] Larimer M.E., Turner A.P., Mallett K.A., Geisner I.M. (2004). Predicting drinking behavior and alcohol-related problems among fraternity and sorority members: examining the role of descriptive and injunctive norms. Psychol. Addict. Behav..

